# The relation between antihypertensive treatment and progression of cerebral small vessel disease: A systematic review and meta-analysis of randomized controlled trials

**DOI:** 10.1097/MD.0000000000026749

**Published:** 2021-07-30

**Authors:** Chen Su, Hao Wu, Xiaoyu Yang, Bing Zhao, Renliang Zhao

**Affiliations:** a Neurology Department, The Affiliated Hospital of Qingdao University, Qingdao, Shandong Province, China; b Neurology Department, Qingdao Municipal Hospital, School of Medicine, Qingdao University, Qingdao, Shandong Province, China.

**Keywords:** antihypertensive treatment, brain atrophy, cerebral small vessel disease, meta-analysis, white matter hyperintensities

## Abstract

**Background:**

Cerebral small vessel disease is relevant to hypertension. We tried to figure out whether antihypertensive treatment is beneficial for this disease.

**Methods:**

We systematically searched PubMed, Embase, and Cochrane electronic databases for randomized controlled trials about white matter hyperintensities (WMH), brain atrophy, microbleeds, and lacunar infarcts with antihypertensive treatment and performed a meta-analysis.

**Results:**

We identified 7 trials on white matter hyperintensities and brain atrophy with antihypertensive treatment. Pooled analysis showed antihypertensive treatment performed positively in the progression of WMH (standardized mean difference, −0.22; 95% CI, −0.36 to −0.07, I^2 = 52%). And in the subgroup meta-analysis, only lower SBP controlled level (110–129 mm Hg) had effect on the progression of WMH (standardized mean difference, −0.37; 95% CI, −0.54 to −0.29, I^2 =0). The meta-regression showed larger difference of SBP in treatment groups having a smaller WMH progression. Antihypertensive treatment is not significant in the progression of brain atrophy (standardized mean difference, −0.02; 95% CI, −0.26 to 0.30, I^2 = 85%). Only 1 trial reported the new patients of lacunar infarcts in the follow-up, no association with antihypertensive treatment (odds ratio, 2.2; 95% CI, 0.4–12.1; *P* = .36).

**Conclusions:**

Antihypertensive treatment is beneficial for cerebral small vessel disease on white matter hyperintensities progression, but no impact on brain atrophy. And lower SBP level is more effective on the progression of WMH. There is not enough evidence to prove the relationship between antihypertensive treatment and lacunar stroke, microbleeds.

## 1 Introduction

Cerebral small vessel disease (cSVD) is a disorder of small cerebral vessels which can cause white matter hyperintensities (WMH), brain atrophy, lacunar infarcts, and cerebral microbleeds (CMBs).^[1,2]^ These lesions are associated with cognitive decline and stroke.^[1]^

Hypertension is an important risk factor for cSVD by producing arteriolosclerosis.^[3]^ And several observational studies have described associations between blood pressure control and cSVD.^[4–6]^ But the outcome of antihypertensive treatment therapy in cSVD is inconsistent.

We aimed to discuss and study the relationship between antihypertensive treatment and progression of cSVD. The randomized controlled trial (RCT) is the most effective way to determine whether an intervention is helpful.^[7]^ So we systematically reviewed and meta-analyzed all RCT evidence on the relation of cSVD progression with antihypertensive treatment. The systematic review and meta-analysis were performed based on based on the Preferred Reporting Items for Systematic reviews and Meta-Analysis (PRISMA) guideline medicine framework population, intervention, comparators, outcomes, study design^[8]^: Did patients with WMH (population) received antihypertensive treatment (intervention) accompanied with no antihypertensive treatment (comparators) have a small progression (outcome) in RCT (study design)?

## 2 Methods

There is no review protocol.

### 2.1 Search strategy

We systematically searched PubMed, Embase, and Cochrane Library for all records published up to September 20, 2018, using the keywords “Antihypertensive,” “hypertension,” “magnetic resonance imaging (MRI),”“ computed tomography (CT),” “white matter,” “infarct,” “lacunes,” “microbleed,” “small vessel,” “brain atrophy.” We also further searched selected publications for relevant complement and contacted with authors to complement the electronic searches. Two independent reviewers assessed all abstracts and full texts, then extracting data from useful articles.

### 2.2 Selection criteria

For the review, we used the following inclusion criteria: all studies had to be RCT of human; cSVD marker (white matter, lacunar infarcts, microbleeds, and brain atrophy) had to be performed on MRI or CT. All studies had to have investigated the association of antihypertensive treatment and progression of cSVD. Interventions may include medications to reduce blood pressure or goals of lowering blood pressure; the control measures were matching placebo or blood pressure control targets. At least 2 years were followed up. We specifically note that we chose WMH and brain atrophy as quantitative outcome measure, given that the changing of lesions can be defined with high precision studies and is less dependent on different assessment criteria. But the lacunar infarcts were measured as qualitative data. To provide additional insight into the association of cSVD with antihypertensive treatment, we also highlighted the associations with difference of SBP in treatment groups as a meta-regression. We restricted our inclusion to original articles that were RCT trails and excluded review articles, case reports, clinical conference papers, and editorials.

### 2.3 Data extraction

We made predefined a data form in which we collected information on characteristics of the study population (age, sex, and inclusion criteria); study design; intervention and control methods; blood pressure levels (baseline, follow-up and change) (Table 1). We also extracted cSVD measurement methods (WMH/brain atrophy/lacunar infarcts, MRI/computed tomography) (Table 2). Outcome assessment and the effect estimate are collected in Table 3.

**Table 1:** Study characteristics of randomized controlled trials investigating relation of antihypertension with cerebral small vessel disease.

Study name	ACCORD-MIND	SPRINT-MIND	PROGRESS CT substudy	PreDIVA	PROGRESS MRI substudy	SCOPE	PRoFESS
Year of publication	2014	2019	2004	2017	2005	2007	2012
Places of participant	North America	North America	Asia	Europe	Europe	Europe, North America, Asia	North and South America, Australia, Asia, Europe
Study design	2 × 2 factorial	Parallel-group	Parallel-group	Parallel-group	Parallel-group	Parallel-group	2 × 2 factorial
intervention	Intensive therapy (SBP <120 mm Hg)	Intensive treatment (SBP <120 mm Hg)	Perindopril 4 mg and indapamide 2 mg	Vascular care (mean ≥2 vascular care visits/yr)	Perindopril 4 mg and indapamide 2.5 mg	Participants aged 70–89 yrs, SBP 160–179 mm Hg and/or DBP 90–99 mm Hg, untreated or thiazidetreated	Telmisartan 80 mg
Control	Standard therapy (SBP <140 mm Hg)	Standard treatment (SBP <140 mm Hg)	Matching placebo	Standard care (mean <2 cross-over vascular care visits/yr)	Placebo	Placebo	Placebo
Inclusion criteria	T2DM at high risk for cardiovascular events, SBP ranging from 130 to 180 mm Hg and taking 3 or fewer antihypertensives	50 yr or older with SBP between 130 and 180 mm Hg at the screening visit and had increased cardiovascular risk	TIA or stroke within the past 5 yrs (excluded subarachnoid hemorrhage)	SBP≥140 mm Hg	TIA or stroke within the past 5 yrs (excluded subarachnoid hemorrhage)	Participants aged 70–89 yrs with SBP 160–179 mm Hg and/or DBP 90–99 mm Hg, untreated or thiazide- treated	An ischemic stroke within the previous 90 d, ≥55 yrs, SBP <180 mm Hg and DBP <110 mm Hg
Time of follow-up (mo)	Mean 40	Median 48 (range 34–57)	Mean 46.8 (sd 1)	Mean 36	Median 36 (range 24–49)	47.3 (sd 0.2)	27.9 (SD 7.6)
Number of participants	314	449	667	126	192	92	771
Age (yrs)	Mean 62.0 (sd 5.4)	Mean 67.1 (sd 7.8)	Mean 64 (sd 9)	Mean 77.2 (sd 8.9)	Mean 60.8 (sd 12.1)	Mean 77 (sd 4)	Mean 65.4 (sd 8.1)
Sex (female)	167 (53.2%)	167 (37.2%)	178 (26.7%)	67 (53.2%)	46 (24.0%)	50 (54.3%)	275 (35.7%)
SBP, intervention							
• baseline	138.7 (sd17.5)	136.0 (sd17.0)	143 (sd 17)	162 (sd 16)	144.3 (sd 20.0)	167 (sd 8)	146.0 (sd 16.3)
• follow-up	118.0 (sd12.0)	122.1 (sd nr)	138 (sd nr)	152 (sd 16)	131.8 (sd nr)	141 (sd 11)	134.9 (sd 20.5)
• change	−20.7	−13.9	−5	−10	−12.5	−26	−11.1
SBP, control							
• baseline	139.3 (sd16.9)	138.2 (sd15.8)	143 (sd 17)	160 (sd 14)	142.2 (sd 19.7)	167 (sd 8)	145.5 (sd 16.3)
• follow-up	133.2 (sd14.6)	136.1 nr	140.4 (sd nr)	156 (sd 15)	140.9 (sd nr)	147 (sd 12)	137.4 (sd 18.2)
• change	−6.1	−2.1	−2.6	−4	−1.3	−20	−8.1
Funding	American Heart Association Scientist Development Grant, NIH-NINDS grants	NIH, including the National Heart, Lung, and Blood Institute, the National Institute of Diabetes and Digestive and Kidney Diseases, the National Institute on Aging, and the National Institute of Neurological Disorders and Stroke	Daiichi Parmaceutical	Dutch Ministry of Health, Innovatiefonds Zorverzekeraars, the Netherlands Organisation for Health Research and Development, Internationale Stichting Alzheimer Onderzoek,	Servier, the Health Research Council of New Zealand and the National Health and Medical Research Council of Australia	AstraZeneca International and Astra Research Foundation UK	Boehringer Ingelheim

**Table 2:** Cerebral small vessel disease determination methods of trails.

Trial	Scan method	Sequences	Field strength	Thickness of slices (mm)	Scan compar- ability	Method of WMH measurement	Method of brain atrophy measurement	Method of lacunar infarction measurement
ACCORD-MIND	MRI	T1, T2, FLAIR, 3D FSPGR	1.5T	1.5–3	Yes	Automatic volumetric measurement	Automatic volumetric measurement	None
SPRINT-MIND	MRI	T1, T2, FLAIR	3T	1	Yes	Lesion segmentation algorithm	Multiatlas label fusion method	None
PROGRESS CT Substudy	CT	None	None	nr	Yes	None	None	Identified by a trained rater on fluid-attenuated inversion recov- ery scans
PreDIVA	MRI	T1,T2	3T	1.2	Yes	k-nearest neighbor algorithm	Adding gray and white matter volumes	nr
PROGRESS MRI Substudy	MRI	T1,T2	1.0T or 1.5T	1.4–5	Yes	A modified version of a validated scale	None	None
SCOPE	MRI	T1, T2, FLAIR	1.5T	1.7–5	Yes	Automated procedure in SPM99	Semiautomated MIDAS	None
PRoFESS	MRI	T1, T2, FLAIR, DWI	nr	nr	nr	Semiquantitative Rotterdam Scan Study scale	nr	None

**Table 3:** Original outcomes of randomized controlled trials included in meta-analysis.

	Brain atrophy							WMH							Lacunar infarction						
	Baseline		Follow-up		Change			Baseline		Follow-up		Change			Baseline		Follow-up		Change		
Trail	Intervention	Control	Intervention	Control	Intervention	Control	Unit	Intervention	Control	Intervention	Control	Intervention	Control	Unit	Intervention	Control	Intervention	Control	Intervention	Control	Unit
ACCORD-MIND	923.7 (sd98.6)	919.3 (sd99.4)	900.7 (sd96.9)	904.9 (sd98.7)	−18.6 (sd16.1)	−14.4 (sd16.6)	cm^3^ (TBV)	2.04 (sd 2.85)	1.80 (sd 2.22)	2.97 (sd 2.77)	2.71 (sd 3.06)	0.67 (sd 0.95)	1.16 (sd 1.13)	cm^3^	None						
SPRINT-MIND	1134.5 (95%CI 1125.1 to 1144.0)	1134.0 (95%CI 1124.4 to 1143.6)	1104.0 (95%CI 1094.5 to 1113.4)	1107.1 (95%CI 1097.4 to 1116.8)	−30.6 (95%CI −32.3 to −28.8)	−26.9 (95%CI −28.8 to −24.9)	cm^3^ (TBV)	4.57 (95%CI 4.00 to 5.14)	4.40 (95%CI 3.80 to 5.00)	5.49 (95%CI 4.91 to 6.07)	5.85 (95%CI 5.23 to 6.47)	0.92 (95%CI 0.69 to 1.14)	1.45 (95%CI 1.21 to 1.70)	cm^3^	None						
PROGRESS CT Substudy	28 (sd 4)	27 (sd 4)	28 (sd 4)	28 (sd 5)	0	1	(cella media index)% of TBV	None							178	169	nr	nr	nr	nr	patient
	33 (sd 4)	33 (sd 5)	33 (sd 5)	33 (sd 6)	0	0	(frontal horn index) % of TBV														
PreDIVA	0.97 (sd 0.10)	0.97 (sd 0.10)	nr	nr	nr	nr	L	6.3 (range 3.5 to 10.9)	5.7 (range 3.3 to 11.1)	nr	nr	0.73 (sd 0.84)	0.70 (sd 0.59)	ml/year	5	4	nr	nr	6	2	Patient
PROGRESS MRI Substudy	None							nr	nr	nr	nr	0.4 (se 0.8)	2.0 (se 0.7)	mm^3^	None						
SCOPE	nr	nr	nr	nr	0.46 (sd 0.42)	0.62 (sd 0.42)	% of TBV	1.09 (sd 1.23)	1.16 (sd 1.39)	1.22 (sd 1.39)	1.34 (sd 1.59)	0.13 (sd 0.30)	0.18 (sd 0.32)	% of TBV	None						
PRoFESS	None							8.17 (sd 6.19)	7.81 (sd 5.86)	8.57 (sd 5.51)	8.71 (sd 6.12)	0.34 (sd 5.45)	0.83 (sd 4.79)	mm (subcortical)	None						
		2.92 (sd 2.31)	2.87 (sd 2.29)	3.48 (sd 2.55)	3.3 (sd 2.46)	0.54 (sd 1.89)	0.40 (sd 1.86)	Score (periventricular)													

### 2.4 Quality assessment

We assessed the quality of the studies using Cochrane risk of bias tool (Table 4). The assessment of cSVD differed considerably across studies, including both qualitative and quantitative measurements and the use of different MRI sequences on which the hyperintensities were quantified. See Table 1 for further details. For the assessment of lacunar infarcts, the criteria were defined by a trained rater on fluid-attenuated inversion recovery scans as round or ovoid, subcortical, fluid-filled (similar signal as cerebral spinal fluid) cavities, between 3 and 15 mm in diameter, regardless of whether these could be linked to any clinical symptoms.^[9]^

**Table 4:**
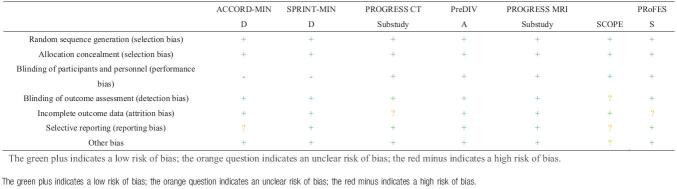
Cochrane risk of bias assessment.

### 2.5 Statistical analyses

We used R Studio to conduct the meta-analysis of antihypertensive treatment with progression of WMH, brain atrophy. Heterogeneity across studies was defined by an I^2 of more than 50%. In this meta-analysis, pooled progression of markers was calculated and performed by standardized mean difference (SMD). Given that preliminary analyses demonstrated considerable heterogeneity across studies, pooled SMD were calculated using random-effects. We performed subgroup analysis to investigate the effective intervention. We also performed a sensitivity analysis examining the comparative outcomes according to the I^2 of more than 50%.

## 3 Results

**Figure 1:**
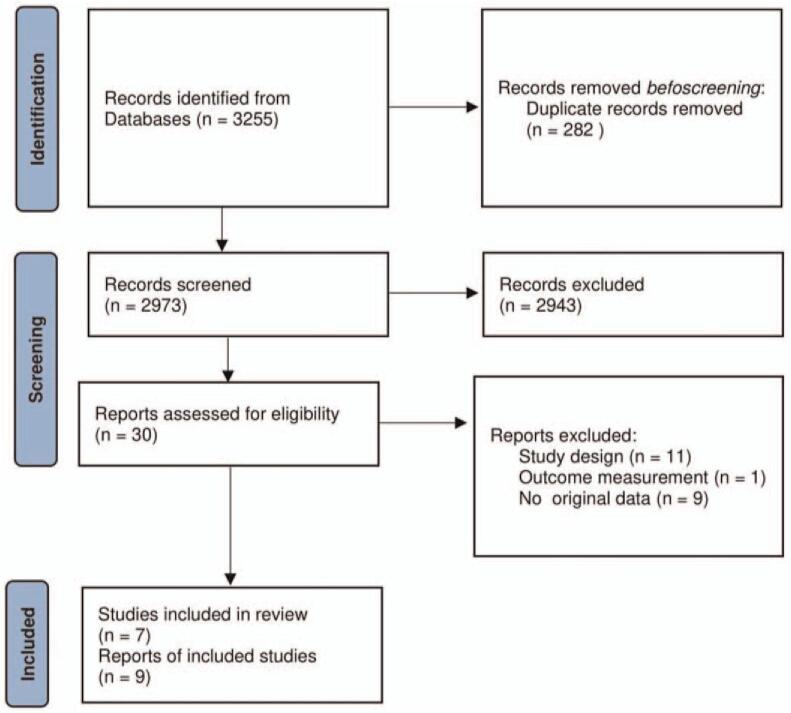
PRISMA flow chart of study selection process. PRISMA = preferred reporting items for systematic reviews and meta-analyses.

We identified 2973 unique articles with the initial search, of which 7 trials were selected finally for meta-analysis (Fig. 1).^[9–17]^ Six trials contained data on WMH with a similar quantitative assessment.^[9–12,14–17]^ Four trials on brain atrophy^[10,12,13,15,17]^ and 1 study on lacunar infarcts.^[9]^ No one study reported CMBs (Table 3).

The total number of participants was 1944, with a mean age ranging from 60 to 78 years at study entry. The inclusions are different, 1 including T2 diabetes patients, ^[10,11]^ 1 including individuals aged 50 years older with high cardiovascular risk, ^[12]^ 1 including people aged 70 to 89 years,^[15]^ 1 including SBP≥140 mm Hg^[9]^ and 3 including patients with stroke or TIA.^[13,14,16]^ 4 studies used placebo as the control measurement,^[13–16]^ 2 studies compared SBP<120 mm Hg to SBP<140 mm Hg,^[10–12]^ and 1 study compared the standard vascular care to intensive vascular care.^[9]^ More information about cSVD measurement methods in Table 1.

**Figure 2:**
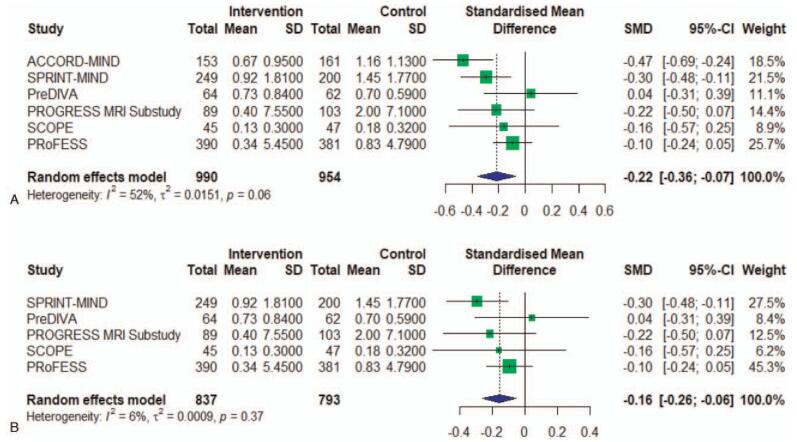
A, Meta-analysis of RCT studies investigating the association of antihypertensive treatment and white matter hyperintensity. RCT = randomized controlled trials. The effect sizes (boxes) with 95% confidence intervals (CI) for the quantitative outcomes are plotted. The size of the box is proportional to the weight of the study. The diamond is the result of the random-effect meta-analysis. B, Meta-analysis of RCT studies investigating the association of antihypertensive treatment and white matter hyperintensity after excluding this study. RCT = randomized controlled trials. The effect sizes (boxes) with 95% confidence intervals (CI) for the quantitative outcomes are plotted. The size of the box is proportional to the weight of the study. The diamond is the result of the random-effect meta-analysis.

**Figure 3:**
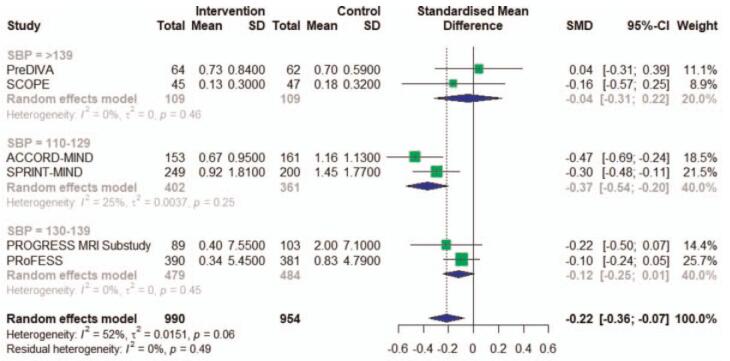
Subgroup meta-analysis of RCT studies investigating the association of antihypertensive treatment and white matter hyperintensity. RCT = randomized controlled trials. The grouping factors are systolic blood pressure in intervention groups at follow-up. The effect sizes (boxes) with 95% confidence intervals (CI) for the quantitative outcomes are plotted. The size of the box is proportional to the weight of the study. The diamond is the result of the random-effect meta-analysis.

**Figure 4:**
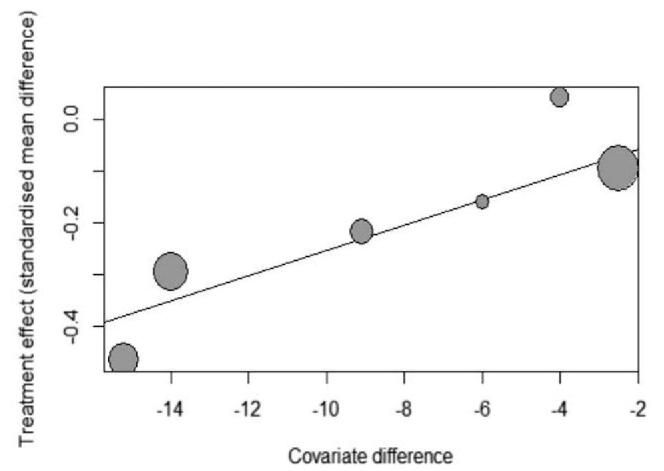
Meta-regression of SBP difference influence on the effect of antihypertensive treatment on WMH progression. Horizontal ordinate means difference of SBP between intervention and control groups; vertical ordinate means WMH progression. SBP = systolic blood pressure, WMH = white matter hyperintensities.

We found a statistically significant difference for the relation between antihypertensive treatment with the progression of WMH of −0.22 (95% CI, −0.36 to −0.07, I^2 = 52%) (Fig. 2A). There obvious heterogeneity between studies (I^2 = 52%), largely accounted for by one single study ^[11]^, that despite having the largest SMD, didn’t obey the blinding of participants and treating physicians. Excluding this study in a sensitivity analysis reduced heterogeneity (I^2 = 6%) and resulted in a pooled SMD of −0.16 (95% CI, −0.26 to −0.06) (Fig. 2B). And in the subgroup meta-analysis, the heterogeneity was low in every group defined by SBP levels in treatment groups, so we found the different SBP intervention level was the source of heterogeneity. The subgroup analysis also showed that SBP level at follow-up impacted the antihypertensive treatment effect on WMH progression. In higher SBP level, antihypertensive treatment had no effect on the progression of WMH. Only the group of 110 to 129 mm Hg showed significant relation with antihypertensive treatment of −0.37 (95% CI, −0.54 to −0.29). The group of 130 to 139 mm Hg of −0.12 (95% CI, −0.25 to −0.01) and the group of >139 mm Hg of −0.04 (95% CI, −0.31 to 0.22) showed no association of antihypertensive treatment with WMH (Fig. 3). We also made a meta-regression of WMH volume changes with differences in SBP between intervention and control at follow-up. And we found larger difference of SBP in treatment groups having a smaller WMH progression (Fig. 4).

**Figure 5:**
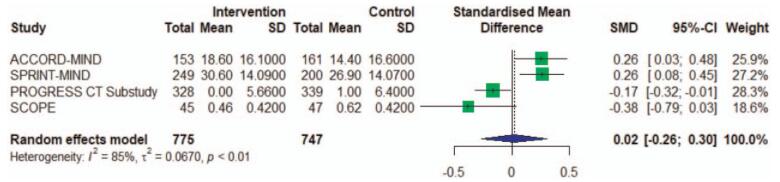
Meta-analysis of RCT studies investigating the association of antihypertensive treatment and brain atrophy. RCT = randomized controlled trials. The effect sizes (boxes) with 95% confidence intervals (CI) for the quantitative outcomes are plotted. The size of the box is proportional to the weight of the study. The diamond is the result of the random-effect meta-analysis.

With 4 studies we found no significant difference in antihypertensive treatment and brain atrophy of 0.02 (95% CI, −0.26 to 0.30, I^2 = 85%) (Fig. 5). The substantial heterogeneity between studies (I^2 = 85%) was difficult to interpretate because the number of included studies is small, possibly caused by different study including conditions, and intervention measures.

One study indicated antihypertensive treatment was no effect on lacunar infarcts, odds ratio of 2.2 (95% confidence interval, 0.4–12.1, *P* = .36).^[9]^

## 4 Discussion

By means of systematic review and meta-analysis of RCT studies on the role of antihypertensive treatment of cVSD, we found evidence that antihypertensive treatment represents an important indicator of a higher effect of preventing progression of WMH. The lower SBP levels seemed to had better effect on stopping WMH progression. But we found no significant relation between antihypertensive treatment and brain atrophy. Only one study reported antihypertensive treatment therapy with no effect on lacunar infarcts incidence, but numbers were low.

Antihypertensive treatment was associated with a decreased risk of WMH progression in the general old-aged population in mean more than 3 years. We also found 1 study in which no association between antihypertensive treatment and WMH volume was reported, but an association between antihypertensive treatment persons with severe WMH load at baseline, the study proportion of participants initiating antihypertensive medication during study was similar in both treatment arms.^[9]^ And another study indicated the higher WMH volume at baseline, the more effective of antihypertensive treatment.^[14]^ In the subgroup meta-analysis, we found the relation of SBP and progression of WMH. Keeping SBP at low level may is more beneficial for prevent WMH from progressing further. The meta-regression also supported the performance, larger difference between treatment groups producing smaller progression, which means lower SBP level in intervention groups can stop the progression. The resultant loss of myelin and gliosis manifests on MRI as WMH.^[18–20]^ The exact mechanism underlying the association of hypertension and WMH is that small cerebral vessels are key targets of hypertension, resulting in pathological alteration of the vascular wall, impairment of vital hemodynamic responses regulating cerebral perfusion, and disruption of blood brain barrier permeability leading to major alterations in the brain microenvironment,^[3]^ and antihypertensive treatment can slow the pathological progression.

Of the above hypertension treatment studies, the ACCORD-MIND trial and SPRINT-MIND trial reported effect on total brain volume (TBV), the intensive blood pressure treatment group showing greater loss of TBV. But PROGRESS CT study and SCOPE trial reported the opposite result. The relationship of hypertension to TBV is less robust and less well documented, although high blood pressure generally has been associated with decreased brain volumes.^[21–23]^ The evidence of lacunar infarcts with antihypertensive treatment is still not enough. Because the number of participants with new lacunar infarcts was too low to allow adjustment in regression analyses, PreDIVA trial did not perform extensive analyses on the outcome and this findings about lacunar infarcts are inconclusive.^[9]^

In terms of the use of antihypertensive drugs, the Progress CT Substudy and the Progress CT Substudy combined Perindopril and Indapamide, while the SCOPE study used Candesartan and the PROESS trial used Telmisartan. The remaining 3 trials did not specify the specific drug to be used (Table 1). Regarding progress in WMH, the results of combined and monotherapy antihypertensive therapy were similar, without statistical correlation with the progress of WMH (Fig. 2). About brain atrophy, the effect of the combination was better than that of the single drug (Fig. 5).

The progression of cSVD is prevalent in patients with hypertension and involved in cognitive impairing as well as an increased risk of stroke, among other consequences.^[24,25]^ And several observational studies have increasingly suggested that cSVD is associated with cognitive decline and the pathogenesis of Alzheimer disease and related dementias.^[26]^ About the mechanism of WMH and dementia, the WMH showed on imaging represents only a tip of the iceberg of the total underlying brain damage, and the composition of WMH varies greatly, ranging from gliosis to demyelination of white matter tracts.^[27–29]^ We found 2 articles reported there no effect of WMH on cognition impairment.^[9,17]^ But 1 article reported participants with probable dementia exhibited significantly larger increases in WMH volume as well as significantly larger decreases in TBV compared with participants having no cognitive impairment.^[12]^ The difference may be caused by the intervention constancy on blood pressure and selective dropout of cognition impairment participants.

Studies have shown that blood pressure variability (BPV) affects cSVD independently of blood pressure levels, and elevated BPV is associated with a higher risk of cSVD.^[30]^ Endothelial cell and blood-brain barrier damage caused by blood pressure fluctuations and perfusion imbalance can induce microglia overactivation, increase the secretion of proinflammatory cytokines and reactive oxygen species, and up-regulation of the neuroinflammatory environment and reactive glial proliferation are considered to be further causes of neurodegenerative changes.^[31]^ The effect of antihypertensive drugs on BPV may modulate the effect of BPV on cSVD, because antihypertensive medications have different effects on the individual blood pressure fluctuations.^[32,33]^ The calcific channel blockers and diuretics are the most effective options for minimizing the BPV.^[34]^ This will give us more help in the selection of antihypertensive drugs on the basis of antihypertensive treatment.

In our meta-analysis, we reviewed and discussed the articles about imaging of antihypertensive treatment and cSVD, but we didn’t put attention on the mechanism and clinical performance of cSVD. The number of included articles was small, so we didn’t conduct calculating publication bias. Blood pressure threshold for therapy initiation, time of treatment, and the blood pressure reduction to maximize benefits and reduce risks are not certain, but the great benefits for general health afforded by blood pressure control justify early and aggressive intervention. More RCT trials processing will reveal the truth of relation between antihypertensive treatment and cSVD finally.^[35,36]^

In conclusion, we found that antihypertensive treatment associated with a decreased progression of WMH, in the general population. And lower SBP level is more effective on the progression of WMH. We found that lacunar infarcts had no relation with antihypertensive treatment. In addition, there was no association between antihypertensive treatment and brain atrophy. No study reported CMBs with antihypertensive treatment. Our results also highlight that RCT data on the association of antihypertensive treatment with cSVD remains limited and that further study into their exact role in the therapy of cSVD is warranted.

## Author contributions

**Conceptualization:** Chen Su, Renliang Zhao.

**Investigation:** Chen Su.

**Resources:** Chen Su, Hao Wu.

**Software:** Su Chen, Hao Wu, Xiaoyu Yang.

**Supervision:** Renliang Zhao.

**Validation:** Renliang Zhao.

**Visualization:** Bing Zhao.

**Writing – original draft:** Chen Su.

**Writing – review & editing:** Chen Su.
